# Based on Network Pharmacology to Explore the Potential Bioactive Compounds and Mechanisms of Zuojin Pill for the Treatment of Ulcerative Colitis

**DOI:** 10.1155/2021/7567025

**Published:** 2021-08-26

**Authors:** Ying Wei, Sichen Ren, Ruilin Wang, Manyi Jing, Honghong Liu, Min Wang, Hongtao Song, Yanling Zhao

**Affiliations:** ^1^School of Pharmacy, Chengdu University of Traditional Chinese Medicine, Chengdu, China; ^2^Department of Pharmacy, The Fifth Medical Center of Chinese PLA General Hospital, Beijing, China; ^3^China Military Institute of Chinese Medicine, The Fifth Medical Center of Chinese PLA General Hospital, Beijing, China; ^4^Department of Pharmacy, 900th Hospital of the Joint Logistics Team, Fuzhou, China

## Abstract

**Background:**

Zuojin Pill (ZJP), a classic prescription, has the potential to prevent ulcerative colitis (UC). However, the active components and mechanisms of ZJP are still arcane. This study aimed to use a network pharmacology approach to find the bioactive compounds and potential action mechanisms of ZJP in the treatment of UC.

**Methods:**

Firstly, the components and putative targets of ZJP were collected based on herbal medicine target databases, and a network containing the interaction between the targets of ZJP and the potential therapeutic targets of UC was established. Then, topological parameters were calculated to identify the key targets in the network and, in turn, to import them into the David database to perform path enrichment analysis.

**Results:**

14 potential therapeutic components of ZJP and 26 key targets were obtained. These targets were related to signal transduction, MAPK cascade, inflammatory response, immune response, and the apoptotic process of UC. Moreover, the PI3K-Akt signaling pathway, MAPK signaling pathway, toll-like receptor signaling pathway, and Prolactin signaling pathway were predicted to participate in ZJP treating UC. Among them, 14 active components of ZJP directly regulate these pathways.

**Conclusion:**

ZJP could alleviate UC through the predicted components and mechanisms. The 14 predicted active components of ZJP may mainly play a therapeutic role for UC through synergistic regulation of the PI3K-Akt signaling pathway and MAPK signaling pathway.

## 1. Introduction

Ulcerative colitis (UC) is a chronic nonspecific inflammatory disease. It was listed as a modern refractory disease by the World Health Organization because of its unclear etiology, high clinical recurrence rate, and association with colon cancer [1]. In recent years, the incidence rate of UC has been increasing worldwide [2]. Aminosalicylic acids, hormones, and immunosuppressants are mainly used in the clinical treatment of UC, but there are obvious adverse reactions. For example, mesalazine is the first-line drug in the treatment of UC, but its long-term application can damage the liver and kidney function of patients [3]. Therefore, many patients with UC as well as physicians and researchers are increasingly considering complementary and alternative medicine options [4, 5].

Zuojin Pill (ZJP), a classic prescription for gastrointestinal diseases, has been widely used in the clinical treatment of gastrointestinal diseases since ancient times in China because of its safety and effectiveness. It composes of *Coptis chinensis* Franch. (Ranunculaceae, recorded in the Chinese Pharmacopoeia as Rhizoma Coptidis) and *Evodia rutaecarpa* (Juss.) Benth. (Rutaceae, recorded in the Chinese Pharmacopoeia as Fructus Evodiae) (6 : 1, g/g).

In recent years, ZJP has been reported to have beneficial evidence on UC [6]. However, the potential active ingredients and molecular mechanism of ZJP on UC are hardly clarified. With the rapid development of bioinformatics, systems biology, and polypharmacology, the pharmacology method based on network pharmacology has been proved to be an effective means to explore the compatibility and mechanisms of traditional Chinese medicine [7–9].

**Figure 1:**
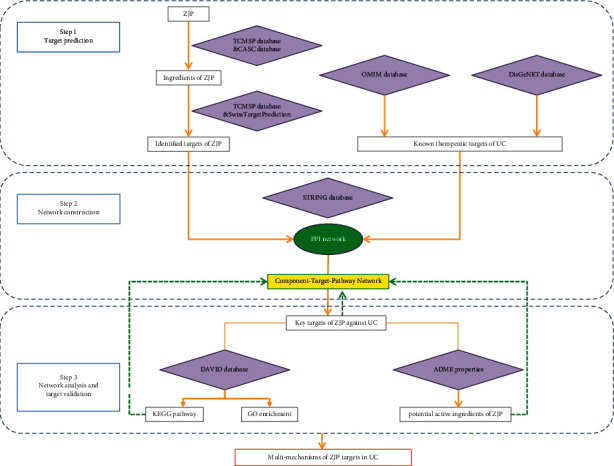
The flowchart of the network pharmacology-based strategy for deciphering the mechanisms of ZJP acting on UC. ZJP: Zuojin pill; UC: ulcerative colitis; TCMSP: Traditional Chinese Medicine Systems Pharmacology Database and Analysis Platform; CASC: Chinese Academy of Sciences Chemistry; OMIM: Online Mendelian Inheritance in Man; STRING: Search Tool for the Retrieval of Interacting Genes/Proteins; PPI: protein-protein interaction; DAVID: the Database for Annotation, Visualization, and Integrated Discovery; KEGG: Kyoto Encyclopedia of Genes and Genomes; GO: gene ontology; ADME: absorption, distribution, metabolism, and excretion.

Therefore, this study aimed to use a comprehensive network pharmacology-based approach to investigate the potential effective components and molecular mechanisms of ZJP in treating UC. The flowchart of the experimental procedures of our study is shown in Figure 1.

## 2. Materials and Methods

### 2.1. Chemical Components of ZJP

We searched the Traditional Chinese Medicine Systems Pharmacology Database and Analysis Platform (TCMSP, https://old.tcmsp-e.com/tcmsp.php, updated on May 31, 2014), Chinese Academy of Sciences Chemistry database (CASC, http://www.organchem.csdb.cn/scdb/main/slogin.asp, updated on December 31, 2019), and related literatures to collect the chemical components of the two herbs contained in ZJP using “Coptidis Rhizoma” and “Fructus Evodiae” as the queries. The TCMSP is a unique systematic pharmacology database of Chinese herbal medicines, chemicals, targets, and drug-target networks. The CASC is one of the most comprehensive chemical databases in the world, which can provide chemical information of Chinese herbal medicine and natural products.

### 2.2. Candidate Targets of ZJP

The targets of chemical components in ZJP were mainly obtained from the TCMSP database. If the targets of chemical constituents reported in the literature were not included in the database, they were obtained through SwissTargetPrediction (http://www.swisstargetprediction.ch/).

### 2.3. Establishment of a Target Database for Treating UC

The known therapeutic targets of UC were acquired from the DisGeNET database (https://www.disgenet.org/) and Online Mendelian Inheritance in Man database (OMIM, https://omim.org/). After screening the target (score ≥ 0.01) and removing the duplicate value, they were collected and used for data analysis serving as a target database for treating UC.

### 2.4. Construction and Analysis of the PPI Network

The protein-protein interactions (PPIs) of each target were generated from the Search Tool for the Retrieval of Interacting Genes/Proteins (STRING) database (https://string-db.org/), and the interactions with a probabilistic association confidence score ≥ 0.9 were selected in this study. All networks were constructed and analyzed by using Cytoscape v3.7.1. Three topological parameters were used as the screening criteria to obtain a hub network and key targets (degree centrality ≥ 2 × median degree centrality; betweenness centrality ≥ median betweenness centrality; and closeness centrality ≥ median closeness centrality).

### 2.5. GO and KEGG Pathway Enrichment Analysis

Gene ontology (GO) analysis and Kyoto Encyclopedia of Genes and Genomes (KEGG) pathway enrichment analysis were performed using the Database for Annotation, Visualization, and Integrated Discovery (DAVID) (https://david.ncifcrf.gov/). GO terms and KEGG pathways with *p* value < 0.05 were considered statistically significant.

### 2.6. Construction and Analysis of the Component-Target-Pathway Network

The component-target-pathway network was constructed and visualized via Cytoscape v3.7.1 to identify key and directly linked molecules and targets. The directly linked molecules were screened by oral bioavailability (OB) and drug-likeness (DL) (OB ≥ 30%, DL ≥ 0.18).

### 2.7. Toxicity Prediction of Components in ZJP

ADMETlab (http://admet.scbdd.com) is a free tool database for the prediction of absorption, distribution, metabolism, and excretion and various toxicities (ADMET) properties and has been widely used in chemical and pharmaceutical fields. Each SMILES of molecule was input to obtain organ toxicity and other toxic properties.

## 3. Results

### 3.1. Composite Ingredients of ZJP

**Figure 2:**
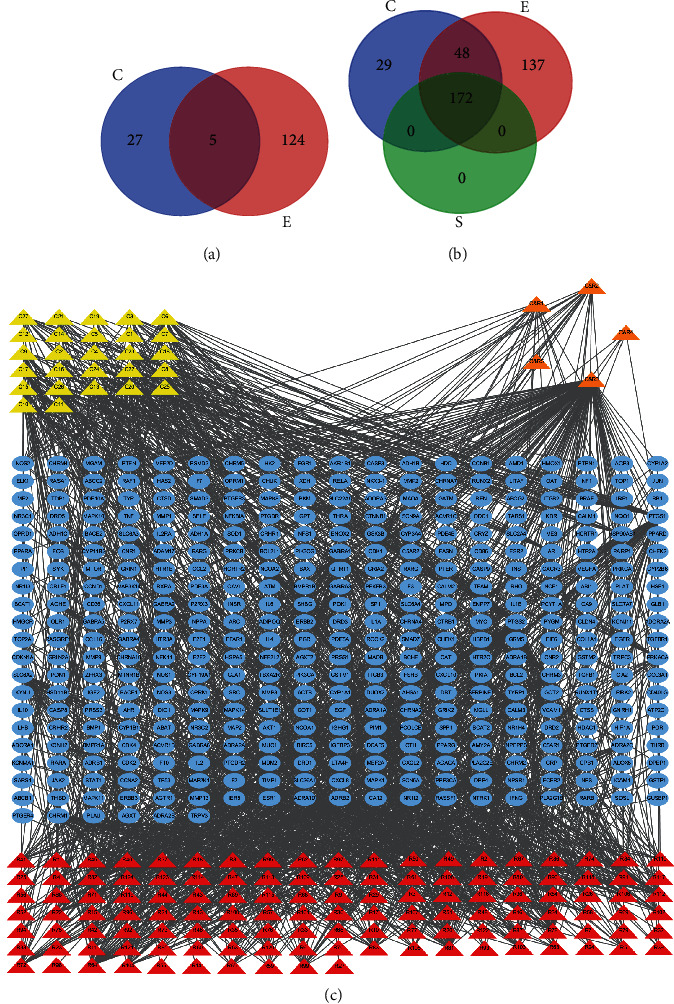
Ingredients and targets of ZJP. (a) Distribution of active compounds among the herbs (C: Rhizoma Coptidis ingredients; E: Fructus Evodiae ingredients). (b) Distribution of potential targets among the herbs (C: Rhizoma Coptidis targets; E: Fructus Evodiae targets; and S: Shared targets). (c) Herb-compound-target network of ZJP (the triangles represent components of ZJP, 32 yellow triangles represent Rhizoma Coptidis components, 127 red triangles represent Rhizoma Coptidis components, 5 orange triangles represent shared components, and blue circles represent the 386 potential targets of ZJP).

A total of 161 chemical ingredients (Table S1) of the two herbal medicines in ZJP were collected from the TCMSP, CASC, and related literatures, including 32 ingredients in Rhizoma Coptidis and 129 ingredients in Fructus Evodiae. There were 5 shared components: berberine, obacunone, quercetin, isovanillin, and limonin (Figure 2(a)).

### 3.2. Putative Targets of Components

The targets of eight components (palmidin A, moupinamide, dehydroevodiamine, evodione, synephrine, obacunone, 13-methylmyristate, and cis-beta-ocimene) were predicted online by SwissTargetPrediction, and the targets of remaining compounds were from the TCMSP. Finally, 249 direct acting targets of Rhizoma Coptidis and 357 direct acting targets of Fructus Evodiae were obtained. After removing repeated targets, 386 targets were obtained for subsequent analysis. Also, there were 220 overlapping targets between the two herbs (Figure 2(b)), which suggested that there might be an interaction between Rhizoma Coptidis and Fructus Evodiae in the treatment process. The relationship between the 161 components and the 386 targets is shown in Figure 2(c).

### 3.3. The Known Therapeutic Targets of UC

**Figure 3:**
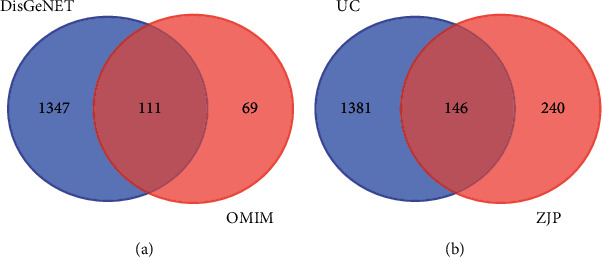
Distribution of the therapeutic targets of UC. (a) Distribution of the known therapeutic targets of UC from two databases. (b) Distribution of ZJP targets and the disease targets. ZJP: Zuojin pill; UC: ulcerative colitis; and OMIM: Online Mendelian Inheritance in Man.

1458 targets of UC were obtained from the DisGeNET database, and 180 targets of UC were obtained from the OMIM database. A total of 1527 targets of UC were obtained after removing the repeats for subsequent analysis (Figure 3(a)). By comparing the components targets and the disease targets through the Venn diagram, 146 targets for the treatment of UC were identified (Figure 3(b)). These targets may be the key proteins of ZJP in the treatment of UC.

### 3.4. The PPI Network of 146 Common Targets

**Figure 4:**
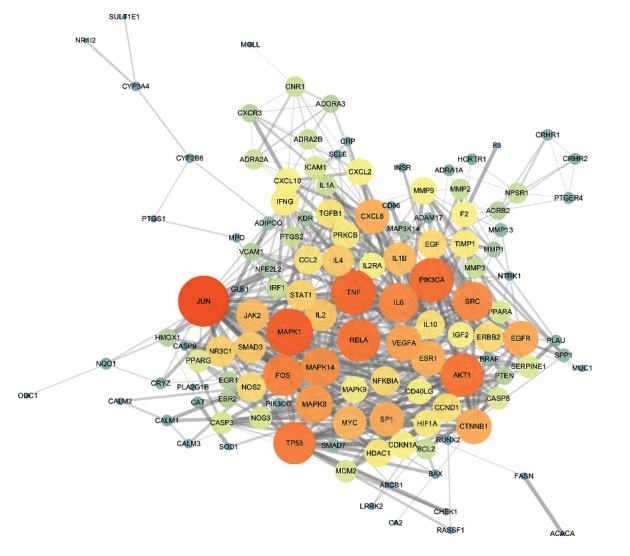
The PPI network of 146 common targets. The higher the degree value is, the brighter the color is and the larger the node is.

The PPI network was constructed by STRING (confidence score ≥ 0.9), with 125 nodes and 645 edges (Figure 4). Based on the topological parameters, we identified 26 key targets, including JUN, MAPK1, TNF, PIK3CA, RELA, AKT1, TP53, FOS, SRC, IL6, MAPK14, MAPK8, VEGFA, CTNNB1, EGFR, SP1, ESR1, CXCL8, MYC, IL1B, JAK2, IL2, IL4, SMAD3, STAT1, and NR3C1 (Table 1).

### 3.5. GO and KEGG Enrichment Analysis

**Figure 5:**
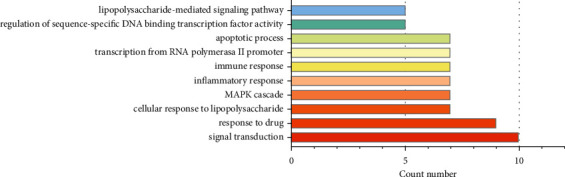
Top 10 GO terms of hub genes.

**Figure 6:**
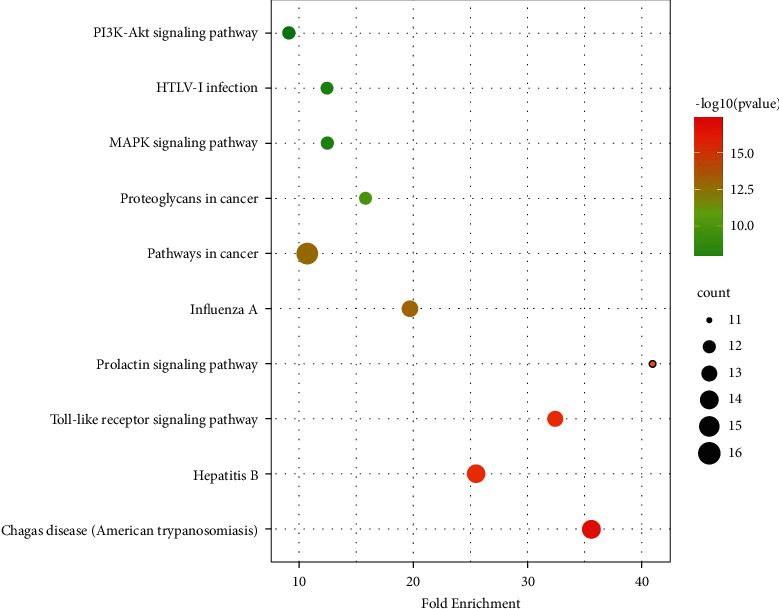
Top 10 KEGG terms of hub genes.

To investigate the biological functions and pathway of the key targets of ZJP, the gene ontology (GO), biological process (BP), and KEGG were performed through the functional annotation tool of DAVID and Cytoscape3.7.1. The top 10 GO terms and pathways were significantly enriched, respectively (Figures 5 and 6 ).

In UC, the BPs mainly regulated by ZJP were signal transduction, response to drug, cellular response to lipopolysaccharide, MAPK cascade, inflammatory response, immune response, transcription from RNA polymerase II promoter, apoptotic process, regulation of sequence-specific DNA binding transcription factor activity, and lipopolysaccharide-mediated signaling pathway (Table 2).

The pathways mainly regulated by ZJP were pathways in cancer, Chagas disease, hepatitis B, the Toll-like receptor signaling pathway, influenza A, proteoglycans in cancer, the MAPK signaling pathway, HTLV-I infection, the PI3K-Akt signaling pathway, and the prolactin signaling pathway (Table 3). Many of these enrichment pathways are related to other pathological effects, which may be due to the similar molecular targets of different diseases. In other words, the same molecular targets may be involved in different pathological processes of diseases. Among them, the changes of the Toll-like receptor signaling pathway, MAPK signaling pathway, and PI3K-Akt signaling pathway are most closely related to UC.

### 3.6. Component-Target-Pathway Network

**Figure 7:**
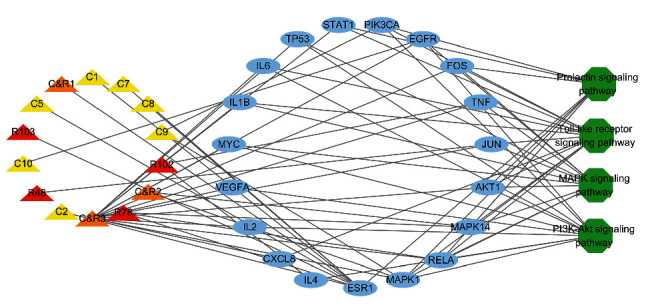
Component-target-pathway network. The triangles represent components of ZJP, 7 yellow triangles represent Rhizoma Coptidis (C) components, red triangles represent Fructus Evodiae (R) components, 3 orange triangles represent shared (C&R) components, 19 blue circles represent the potential targets of ZJP, and 4 green octagons represent UC-related signaling pathways.

The key targets of ZJP enriched to four signaling pathways are shown in Table 3. We constructed the “component-target-pathway” network with 14 directly linked components screened by ADME, 19 directly regulated targets, and four UC-related signaling pathways (Figure 7). The network showed that these 14 molecules were involved in regulating the toll-like receptor signaling pathway, MAPK signaling pathway, prolactin signaling pathway, and PI3K-Akt signaling pathway. In this network, we identified directed molecules and targets (Table 4). In order to further characterize the safety of these active ingredients, toxicity prediction of 14 active components in ZJP is shown in Table 5.

## 4. Discussion

The pathogenesis of UC is sophisticated, including genetic, environmental, psychological stress, and other factors [1]. In view of the complicated pathogenesis, a single target drug hardly obtains better therapeutic effect, while traditional Chinese medicine (TCM) with multitarget effect has become a considerable source of UC therapeutic drugs [10, 11]. Moreover, network pharmacology provides a new strategy for the research of TCM through exploring the relationship between drugs and diseases from a holistic perspective.

In this study, we successfully identified 14 active molecules and 3 important pathways related to UC through network pharmacology and bioinformatics. This method can more clearly identify the targets and specific mechanisms of TCM in the treatment of diseases and obtain the key active molecules, which is of great significance for the development of natural drugs and the treatment of diseases.

**Figure 8:**
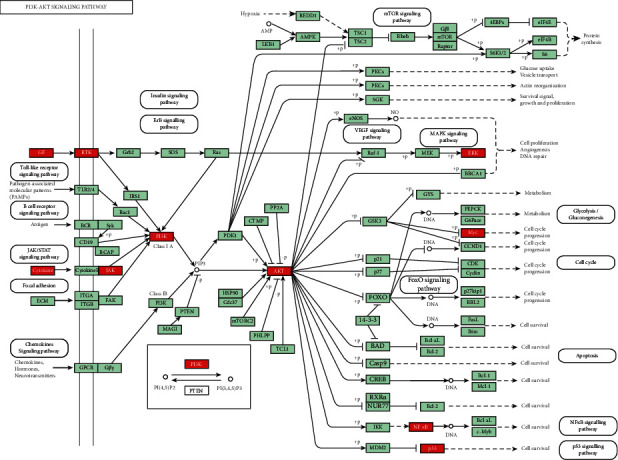
PI3K-Akt signaling pathway (KEGG). Red nodes represent the targets regulated by ZJP in UC.

**Figure 9:**
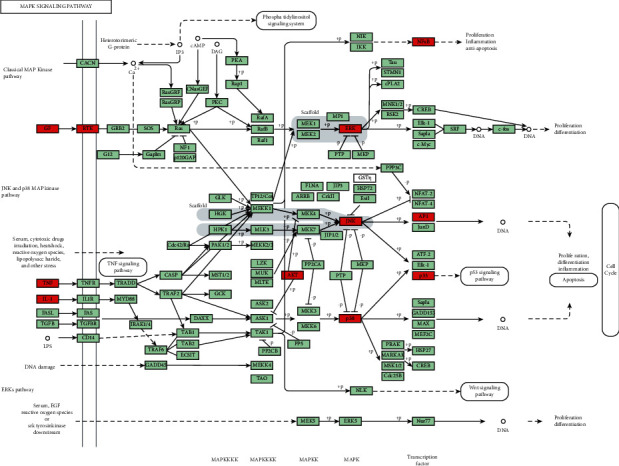
MAPK signaling pathway (KEGG). Red nodes represent the targets regulated by ZJP in UC.

**Figure 10:**
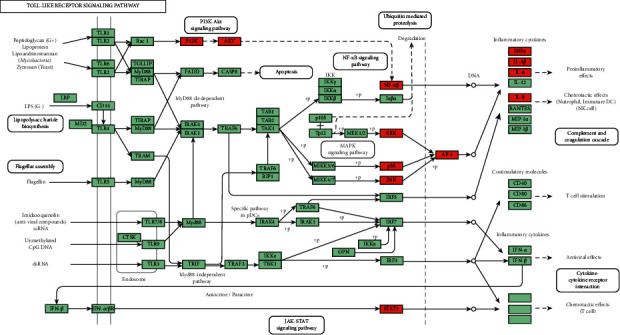
Toll-like receptor signaling pathway (KEGG). Red nodes represent the targets regulated by ZJP in UC.

GO-BP enrichment analysis showed that ZJP may play an anti-UC role by regulating inflammatory response, immune response, apoptosis, MAPK cascade, signal transduction, and other biological functions. Pathway enrichment analysis results showed that many other diseases were enriched in addition to UC, which may be because the same molecular targets exist in different pathological processes of diseases. This is not only the limitation of network pharmacology but also the divergence points of the research direction. Therefore, we selected the enrichment pathway closely related to UC in our research. Finally, based on the two aspects of network construction and central network evaluation, 26 key targets of the ZJP potential target network were selected which were significantly enriched in three UC-related signaling pathways, including the PI3K-Akt signaling pathway, MAPK signaling pathway, and toll-like receptor signaling pathway. Studies have shown that the PI3K-Akt signaling pathway could regulate the inflammation and oxidative stress of UC [12, 13]; the MAPK pathway played a key role in regulating the development of UC inflammation [14]; the toll-like receptor signaling pathway played an important role in the immune system and participated in the inflammatory process of UC [15]. However, the key target of this study enriched in the toll-like receptor signaling pathway was focused on the downstream MAPK pathway crosstalk. Thereby, it could be considered that ZJP treatment of UC mainly directly regulated the PI3K-Akt signaling pathway and MAPK signaling pathway. Figures 8–10 summarize the representative pathways in the progress of UC and the key targets (red nodes) related to ZJP, indicating that ZJP treatment of UC may be closely related to the regulation of inflammation and cell proliferation.

Among the 14 predicted active components, 9 compounds such as berberine, obacunone, quercetin, coptisine derivatives, berberrubine, palmatine, beta sitosterol, isorhamnetin, and rutaecarpine have been proved to be effective in the treatment of UC, and the related mechanism of some compounds was consistent with the predicted pathway in this study. Studies have shown that berberine could inhibit the p38 MAPK signaling pathway and decrease the levels of inflammatory biomarkers IL-1, TNF-*α*, and IL-6 in UC [16]; obacunone could protect against UC in mice by modulating gut microbiota, attenuating TLR4/NF-*κ*B signaling cascades, and improving disrupted epithelial barriers [17]; quercetin could inhibit the release of proinflammatory mediators and the expression of inflammatory proteins in UC [18]; coptisine derivatives such as dihydrocoptisine, quaternary coptisines, and tetrahydrocoptisines were proved to have anti-UC activities, among which dihydroberberine has better development value [19]; berberrubine could attenuate mucosal lesions and inflammation in UC [20]; palmatine could attenuate dextran sulfate sodium- (DSS-) induced colitis via promoting mitophagy-mediated NLRP3 inflammasome inactivation [21]; *β*-Sitosterol could improve experimental colitis in mice by increasing the expression of antimicrobial peptides in intestinal epithelial cells [22]; isorhamnetin could ameliorate experimental IBD via PXR-mediated upregulation of xenobiotic metabolism and downregulation of NF-*κ*B signaling [23]; rutaecarpine could improve experimental colitis by regulating the synthesis and release of CGRP [24]. In general, most of these active ingredients could exert an anti-inflammatory response, regulate intestinal flora, and maintain the integrity of the mucosal epithelial barrier through the PI3K-Akt signaling pathway, MAPK signaling pathway, and toll-like receptor signaling pathway predicted in this study.

In addition to the abovementioned active ingredients with the research basis related to UC, five potential active ingredients including worenine, epiberberine, palmidin A, moupinamide, and rutalinidine have not been reported in UC. Recent studies have shown that worenine could reverse the Warburg effect and inhibit colon cancer cell growth by negatively regulating HIF-1*α* [25]; epiberberine could be a novel antitumor candidate against MKN-45-related gastric cancer via targeting the p53-dependent mitochondria-associated pathway [26]; moupinamide had anti-inflammatory potential found in Aswad's study [27]. Furthermore, there are few studies on palmidin A and rutalinidine, and the toxicity of rutalinidine was predicted to be low in this study, which was more worthy of further study. Precisely, because the effects and mechanisms of these potential active ingredients on UC have not been explained and verified, the research space and value are enormous. The abovementioned evidence preliminarily has suggested that these components of ZJP obtained by network pharmacology analysis may be effective components and jointly play anti-UC roles. These may be the material basis of ZJP in the treatment of UC, but further experimental verification and mechanism research are needed.

## 5. Conclusions

In short, researching ZJP on UC by use of the network pharmacological approach reflected the multicomponent, multitarget, and integrated regulation of TCM prescriptions. This study predicted the potential active ingredients, molecular targets, and mechanisms of ZJP in the treatment of UC, which provided a prospective scientific reference for the future systematic study of the effects and mechanisms of ZJP and its material basis on UC.

## Figures and Tables

**Table 1:** Hub genes in the PPI network.

Gene name	Degree	Betweenness centrality	Closeness centrality
JUN	40	0.0866053	0.52765957
MAPK1	35	0.10522212	0.51239669
TNF	33	0.06737271	0.49206349
PIK3CA	32	0.09809998	0.49011858
RELA	31	0.04010596	0.50612245
AKT1	31	0.07469638	0.49011858
TP53	30	0.06701158	0.49011858
FOS	28	0.03619664	0.484375
SRC	27	0.05381998	0.496
IL6	27	0.05383839	0.484375
MAPK14	26	0.02930984	0.47328244
MAPK8	24	0.0236753	0.46096654
VEGFA	23	0.05077093	0.5
CTNNB1	22	0.03781139	0.46268657
EGFR	22	0.05992162	0.48627451
SP1	21	0.03912481	0.46441948
ESR1	21	0.00980253	0.47148289
CXCL8	21	0.06791767	0.44604317
MYC	20	0.00939055	0.46969697
IL1B	19	0.01687947	0.46441948
JAK2	19	0.01407263	0.45925926
IL2	18	0.00668683	0.45588235
IL4	17	0.01066433	0.44765343
SMAD3	17	0.01141516	0.43055556
STAT1	16	0.01339176	0.44927536
NR3C1	16	0.00783583	0.44765343

**Table 2:** Top 10 GO terms of hub genes.

Term	Count	%	*p* value
Signal transduction	10	38.46	2.60E-05
Response to drug	9	34.62	8.71E-09
Cellular response to lipopolysaccharide	7	26.92	1.30E-08
MAPK cascade	7	26.92	1.88E-06
Inflammatory response	7	26.92	1.56E-05
Immune response	7	26.92	2.83E-05
Transcription from RNA polymerase II promoter	7	26.92	8.52E-05
Apoptotic process	7	26.92	1.48E-04
Regulation of sequence-specific DNA binding transcription factor activity	5	19.23	4.73E-08
Lipopolysaccharide-mediated signaling pathway	5	19.23	1.34E-07

**Table 3:** Top 10 KEGG pathways of hub genes.

Term	Count	%	*p* value	Genes
Pathways in cancer	16	61.54	3.36E-13	JUN, SMAD3, CXCL8, STAT1, FOS, EGFR, RELA, VEGFA, IL6, MAPK8, PIK3CA, MYC, AKT1, MAPK1, CTNNB1, and TP53
Chagas disease	14	53.85	4.42E-18	JUN, SMAD3, CXCL8, FOS, MAPK14, TNF, IL2, RELA, IL6, MAPK8, PIK3CA, IL1B, AKT1, and MAPK1
Hepatitis B	14	53.85	3.88E-16	JUN, CXCL8, STAT1, SRC, FOS, TNF, RELA, IL6, MAPK8, PIK3CA, MYC, AKT1, MAPK1, and TP53
Toll-like receptor signaling pathway	13	50.00	4.12E-16	JUN, CXCL8, STAT1, FOS, MAPK14, TNF, RELA, IL6, MAPK8, PIK3CA, IL1B, AKT1, and MAPK1
Influenza A	13	50.00	1.84E-13	JUN, CXCL8, STAT1, MAPK14, TNF, RELA, IL6, MAPK8, PIK3CA, IL1B, AKT1, MAPK1, and JAK2
Proteoglycans in cancer	12	46.15	2.98E-11	PIK3CA, SRC, MYC, MAPK1, CTNNB1, AKT1, MAPK14, ESR1, TNF, TP53, EGFR, and VEGFA
MAPK signaling pathway	12	46.15	3.79E-10	JUN, MAPK8, MYC, IL1B, MAPK1, AKT1, FOS, MAPK14, TNF, TP53, RELA, EGFR
HTLV-I infection	12	46.15	3.96E-10	IL6, JUN, SMAD3, PIK3CA, MYC, CTNNB1, AKT1, FOS, TNF, TP53, RELA, and IL2
PI3K-Akt signaling pathway	12	46.15	1.02E-08	IL4, IL6, PIK3CA, MYC, MAPK1, AKT1, JAK2, TP53, RELA, EGFR, IL2, and VEGFA
Prolactin signaling pathway	11	42.31	2.05E-14	MAPK8, PIK3CA, STAT1, SRC, MAPK1, AKT1, FOS, JAK2, MAPK14, ESR1, and RELA

**Table 4:** Directly linked components screened by OB and DL.

Code name	Molecule name	MW	OB (%)	DL
C&R1	Berberine	336.39	36.86	0.78
C&R2	Obacunone	454.56	43.29	0.77
C&R3	Quercetin	302.25	46.43	0.28
C1	Worenine	334.37	45.83	0.87
C2	Coptisine	320.34	30.67	0.86
C5	Epiberberine	336.39	43.09	0.78
C7	Berberrubine	322.36	35.74	0.73
C8	Palmatine	510.52	35.36	0.65
C9	Palmidin A	510.52	35.36	0.65
C10	Moupinamide	313.38	86.71	0.26
R48	Beta-sitosterol	414.79	36.91	0.75
R78	Isorhamnetin	316.28	49.6	0.31
R102	Rutaecarpine	287.34	40.3	0.6
R103	Rutalinidine	275.33	40.89	0.22

Abbreviations: C: Rhizoma Coptidis components; R: Fructus Evodiae components; C&R: Rhizoma Coptidis and Fructus Evodiae shared components.

**Table 5:** Toxicity prediction of 14 active components in ZJP.

Code name	Molecule name	Ames mutagenesis	Hepatotoxicity	Acute oral toxicity	Acute oral toxicity evaluation
CR1	Berberine	−−−	++	551.84 mg/kg	Low
CR2	Obacunone	−−	+	51.827 mg/kg	Toxicity
CR3	Quercetin	++	+	698.794 mg/kg	Low
C1	Worenine	−	+	558.731 mg/kg	Low
C2	Coptisine	−	−	547.759 mg/kg	Low
C5	Epiberberine	−−−	++	571.233 mg/kg	Low
C7	Berberrubine	−−−	++	301.519 mg/kg	Toxicity
C8	Palmatine	−	++	660.767 mg/kg	Low
C9	Palmidin A	+	−−−	147.569 mg/kg	Toxicity
C10	Moupinamide	−	++	1603.37 mg/kg	Low
R48	Beta-sitosterol	−−−	−−−	273.371 mg/kg	Toxicity
R78	Isorhamnetin	−−−	+	604.02 mg/kg	Low
R102	Rutaecarpine	−−−	++	624.265 mg/kg	Low
R103	Rutalinidine	−	+++	591.306 mg/kg	Low

*Note.* The “+” and “−” represent the predicted toxicity possibility. 0.1(−−−); 01–0.3(−−); 0.3–05(−); 0.5–0.7(+); 0.7–0.9(++); 0.9–10(+++). Acute oral toxicity evaluation involves high toxicity (1∼50 mg/kg), toxicity (51∼500 mg/kg), and low toxicity (501∼5000 mg/kg). C: Rhizoma Coptidis components; R: Fructus Evodiae components; C&R: Rhizoma Coptidis and Fructus Evodiae shared components.

## Data Availability

All data are available in the manuscript, and they are shown in figures and tables.

## Abbreviations

ZJP: Zuojin pill
UC: Ulcerative colitis
TCMSP: Traditional Chinese Medicine Systems Pharmacology Database and Analysis Platform
CASC: Chinese Academy of Sciences Chemistry
OMIM: Online Mendelian Inheritance in Man
PPI: Protein-protein interactions
DAVID: The Database for Annotation, Visualization, and Integrated Discovery
KEGG: Kyoto Encyclopedia of Genes and Genomes
GO: Gene Ontology
ADME: Absorption, distribution, metabolism, and excretion
STRING: Search Tool for the Retrieval of Interacting Genes/Proteins
DL: Drug-likeness
OB: Oral bioavailability
BP: Biological process.

## Supplementary Materials

Supplementary Materials Table S1. Ingredients of each herb contained in ZJP. .
