# Supplementary material for: Based on Network Pharmacology to Explore the Potential Bioactive Compounds and Mechanisms of Zuojin Pill for the Treatment of Ulcerative Colitis
Source: Evid Based Complement Alternat Med. 2021 Aug 26;2021:7567025. doi: 10.1155/2021/7567025 (PMC8416371; doi:10.1155/2021/7567025)
Supplement: Supplementary Materials — Table S1. Ingredients of each herb contained in ZJP. . [file 7567025.f1.docx]

| Table S1. Ingredients of each herb contained in ZJP | | | | | | |
| --- | --- | --- | --- | --- | --- | --- |
| Herb | Number | Code  name | Compound | Pubchem ID | Molecular formula | Structure |
| Coptidis Rhizoma | 1 | C1 | Worenine | 20055073 | C20H16NO4+ | 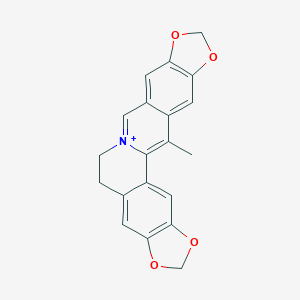 |
| Coptidis Rhizoma | 2 | C2 | coptisine | 72322 | C19H14NO4+ | 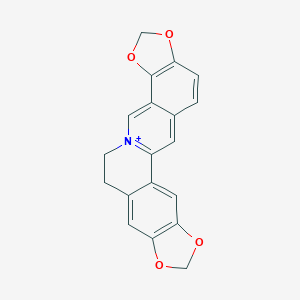 |
| Coptidis Rhizoma | 3 | C3 | Berlambine | 11066 | C20H17NO5 | 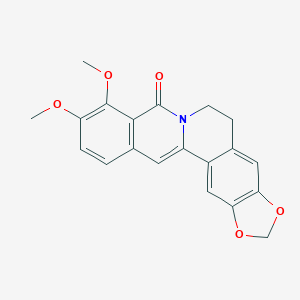 |

| Coptidis Rhizoma | 4 | C4 | orchoroside A_ | 5091219 | C29H42O9 | 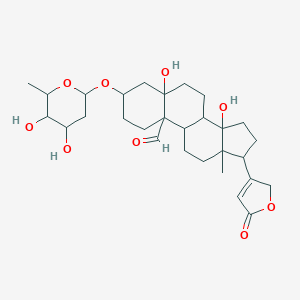 |
| --- | --- | --- | --- | --- | --- | --- |
| Coptidis Rhizoma | 5 | C5 | epiberberine | 160876 | C20H18NO4+ | 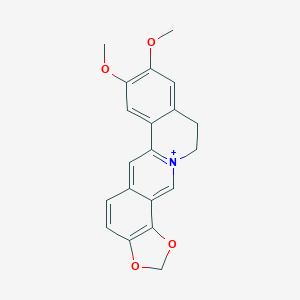 |
| Coptidis Rhizoma | 6 | CR1 | berberine | 2353 | C20H18NO4+ | 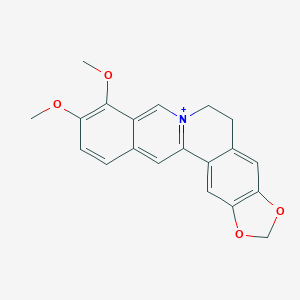 |

| Coptidis Rhizoma | 7 | C6 | (R)-Canadine | 443422 | C20H21NO4 | 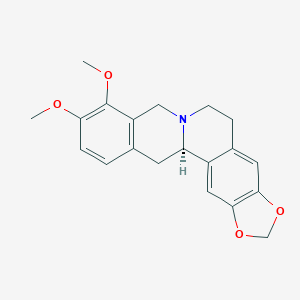 |
| --- | --- | --- | --- | --- | --- | --- |
| Coptidis Rhizoma | 8 | CR2 | Obacunone | 119041 | C26H30O7 | 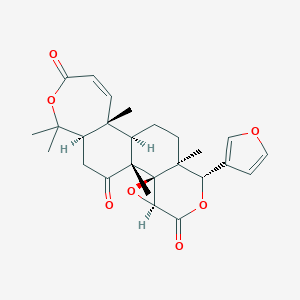 |
| Coptidis Rhizoma | 9 | C7 | berberrubine | 72703 | C19H16ClNO4 | 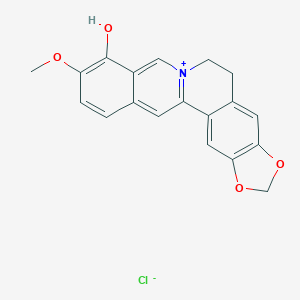 |

| Coptidis Rhizoma | 10 | C8 | palmatine | 19009 | C21H22NO4+ | 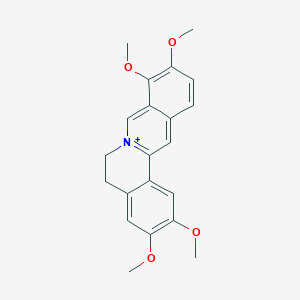 |
| --- | --- | --- | --- | --- | --- | --- |
| Coptidis Rhizoma | 11 | C9 | Palmidin A | 5320384 | C30H22O8 | 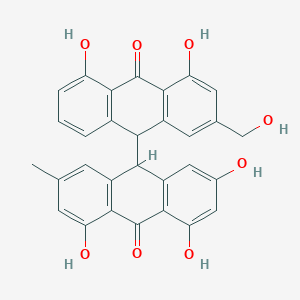 |
| Coptidis Rhizoma | 12 | CR3 | quercetin | 5280343 | C15H10O7 | 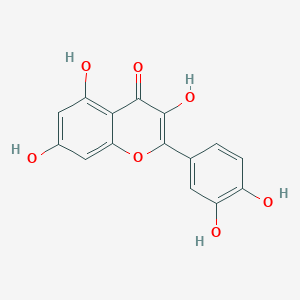 |

| Coptidis Rhizoma | 13 | C10 | Moupinamide | 5280537 | C18H19NO4 | 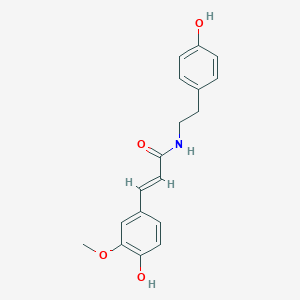 |
| --- | --- | --- | --- | --- | --- | --- |
| Coptidis Rhizoma | 14 | C11 | agnograndioli | 5319198 | C15H22O4 | 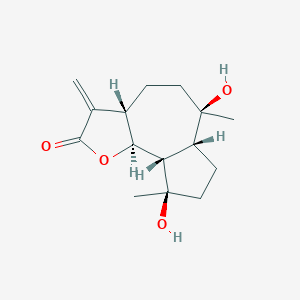 |
| Coptidis Rhizoma | 15 | C12 | Fagarine | 107936 | C13H11NO3 | 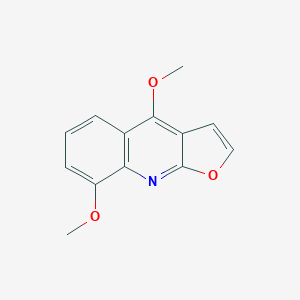 |

| Coptidis Rhizoma | 16 | C13 | Corydaldine | 610097 | C11H13NO3 | 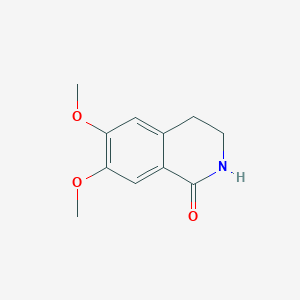 |
| --- | --- | --- | --- | --- | --- | --- |
| Coptidis Rhizoma | 17 | C14 | Ethyl caffeate | 5317238 | C11H12O4 | 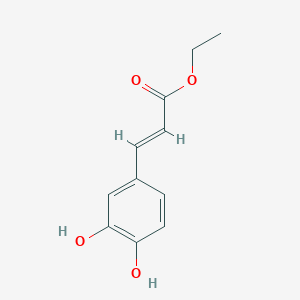 |
| Coptidis Rhizoma | 18 | C15 | hydroxytyroso | 82755 | C8H10O3 | 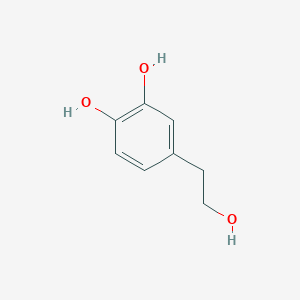 |

| Coptidis Rhizoma | 19 | C16 | -coumaric aci | 637542 | C9H8O3 | 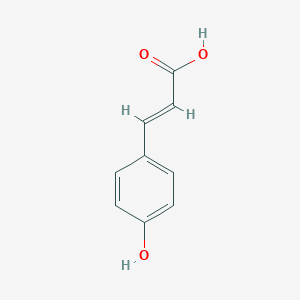 |
| --- | --- | --- | --- | --- | --- | --- |
| Coptidis Rhizoma | 20 | C17 | danshensu | 11600642 | C9H10O5 | 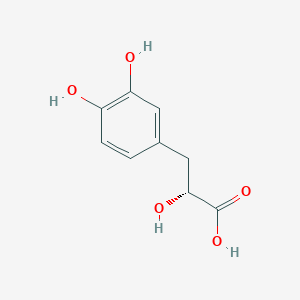 |
| Coptidis Rhizoma | 21 | C18 | vanillic acid | 8468 | C8H8O4 | 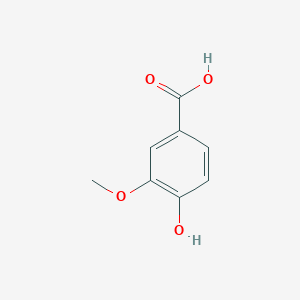 |

| Coptidis Rhizoma | 22 | CR4 | Isovanillin | 12127 | C8H8O3 | 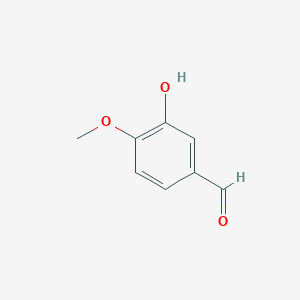 |
| --- | --- | --- | --- | --- | --- | --- |
| Coptidis Rhizoma | 23 | C19 | roxyhydrastin | 89047 | C10H9NO3 | 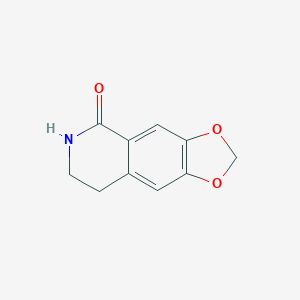 |
| Coptidis Rhizoma | 24 | C20 | columbamine | 72310 | C20H20NO4+ | 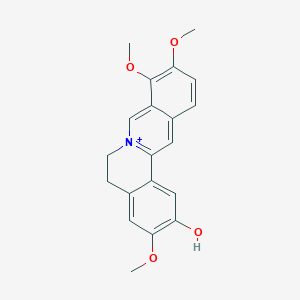 |

| Coptidis Rhizoma | 25 | C21 | Tetrandrine | 73078 | C38H42N2O6 | 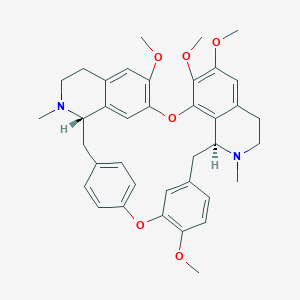 |
| --- | --- | --- | --- | --- | --- | --- |
| Coptidis Rhizoma | 26 | CR5 | limonin | 179651 | C26H30O8 | 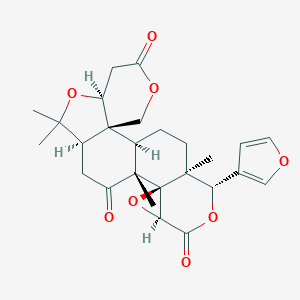 |
| Coptidis Rhizoma | 27 | C22 | Magnoflorine | 73337 | C20H24NO4+ | 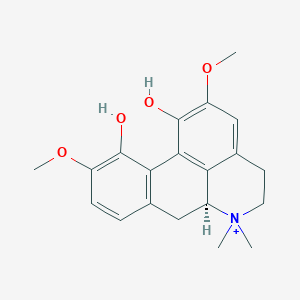 |

| Coptidis Rhizoma | 28 | C23 | phellodendrine | 3081405 | C20H24NO4+ | 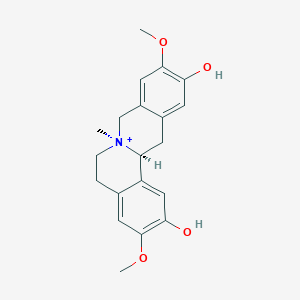 |
| --- | --- | --- | --- | --- | --- | --- |
| Coptidis Rhizoma | 29 | C24 | Zosimin | 6436246 | C19H20O5 | 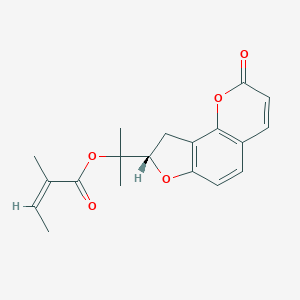 |
| Coptidis Rhizoma | 30 | C25 | groenlandicine | 3084708 | C19H16NO4+ | 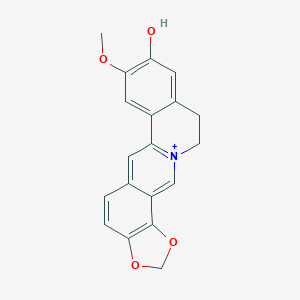 |

| Coptidis Rhizoma | 31 | C26 | FER | 445858 | C10H10O4 | 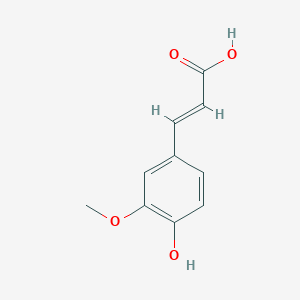 |
| --- | --- | --- | --- | --- | --- | --- |
| Coptidis Rhizoma | 32 | C27 | jatrorrhizine | 72323 | C20H20NO4+ | 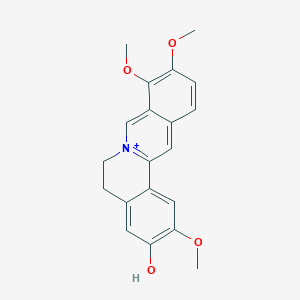 |
| Evodiae Fructus | 33 | R1 | -)-alpha-Pinen | 440968 | C10H16 | 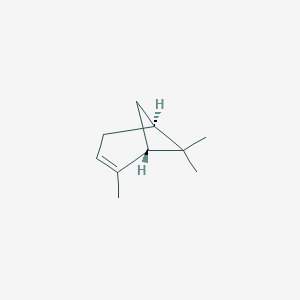 |

| Evodiae Fructus | 34 | R2 | aryophyllene | 1742210 | C15H24O | 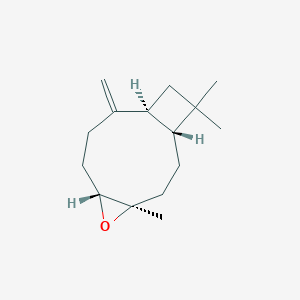 |
| --- | --- | --- | --- | --- | --- | --- |
| Evodiae Fructus | 35 | R3 | )-Terpinen-4-o | 2724161 | C10H18O | 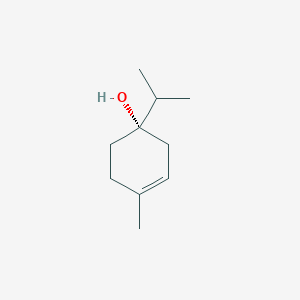 |
| Evodiae Fructus | 36 | R4 | beta-Phelland | 442484 | C10H16 | 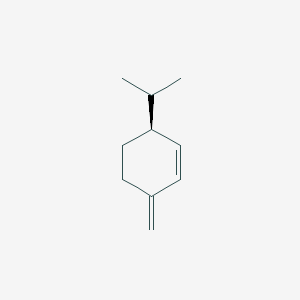 |

| Evodiae Fructus | 37 | R5 | -methylpent-3 | 13889654 | C15H24 | 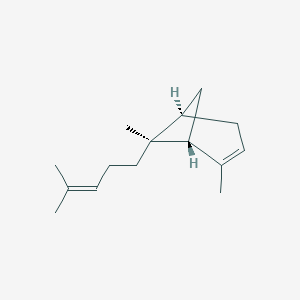 |
| --- | --- | --- | --- | --- | --- | --- |
| Evodiae Fructus | 38 | R6 | -4-methyleneb | 11051711 | C10H16 | 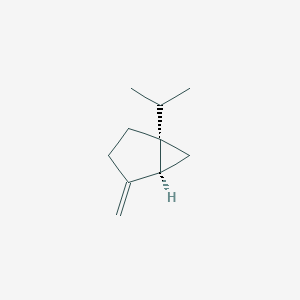 |
| Evodiae Fructus | 39 | R7 | ramethylhexad | 11335274 | C20H34O | 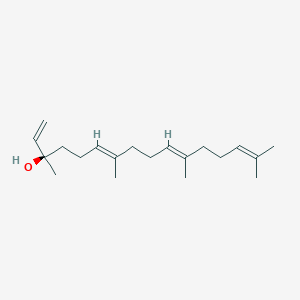 |

| Evodiae Fructus | 40 | R8 | y-6-isopropeny | 10419566 | C10H16O2 | 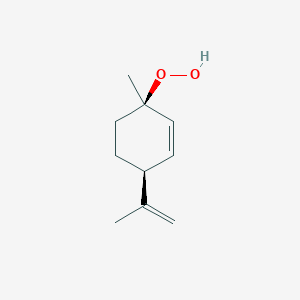 |
| --- | --- | --- | --- | --- | --- | --- |
| Evodiae Fructus | 41 | R9 | ethyl-2,3-dihy | 11423556 | C5H8O | 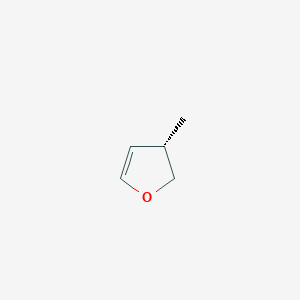 |
| Evodiae Fructus | 42 | R10 | ylcyclohexene- | 11842593 | C10H16O | 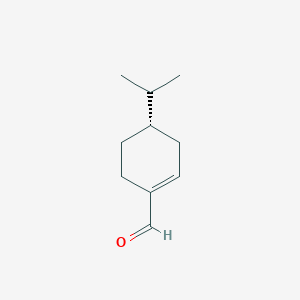 |

| Evodiae Fructus | 43 | R11 | (R)-linalool | 443158 | C10H18O | 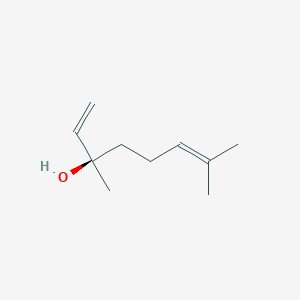 |
| --- | --- | --- | --- | --- | --- | --- |
| Evodiae Fructus | 44 | R12 | )-alpha-Phella | 443160 | C10H16 | 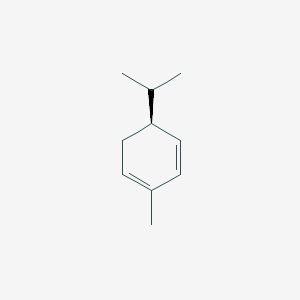 |
| Evodiae Fructus | 45 | R13 | )-caryophylle | 6429301 | C15H24 | 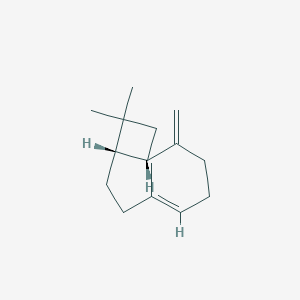 |

| Evodiae Fructus | 46 | R14 | imethyloct-6-e | 6999975 | C12H22O2 | 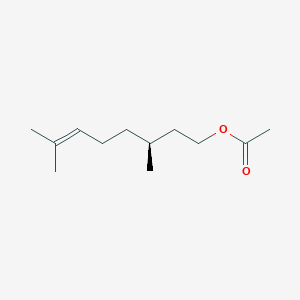 |
| --- | --- | --- | --- | --- | --- | --- |
| Evodiae Fructus | 47 | R15 | 2-enyl](Z)-3-p | 1550888 | C18H16O2 | 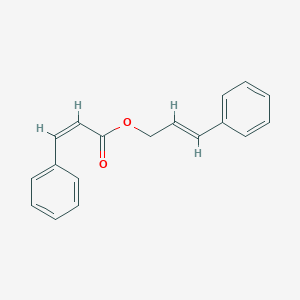 |
| Evodiae Fructus | 48 | R16 | 2,2-dimethylch | 624052 | C16H20O5 | 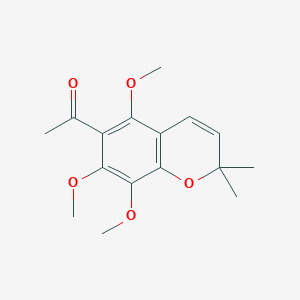 |

| Evodiae Fructus | 49 | R17 | rimethylcyclo | 136316 | C8H14 | 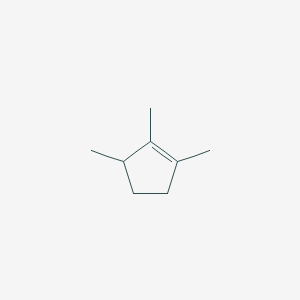 |
| --- | --- | --- | --- | --- | --- | --- |
| Evodiae Fructus | 50 | R18 | ,8-p-Menthatri | 176983 | C10H14 | 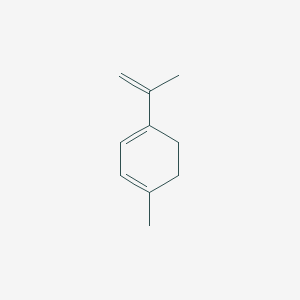 |
| Evodiae Fructus | 51 | R19 | pentadec-10-e | 5319752 | C25H37NO | 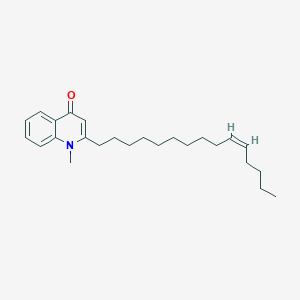 |

| Evodiae Fructus | 52 | R20 | l-2-nonyl-4-qu | 13967189 | C19H27NO | 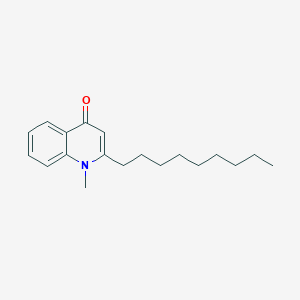 |
| --- | --- | --- | --- | --- | --- | --- |
| Evodiae Fructus | 53 | R21 | -pentadecyl-4 | 5319753 | C25H39NO | 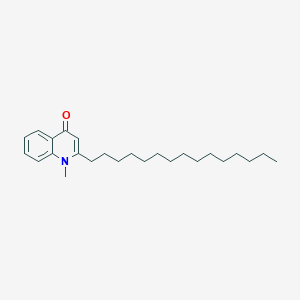 |
| Evodiae Fructus | 54 | R22 | -2-undecyl-4-q | 5319811 | C21H31NO | 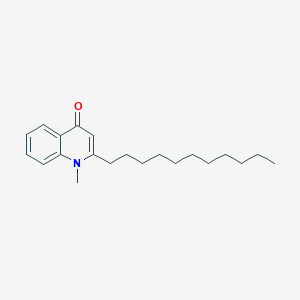 |

| Evodiae Fructus | 55 | R23 | 1-Penten-3-on | 15394 | C5H8O | 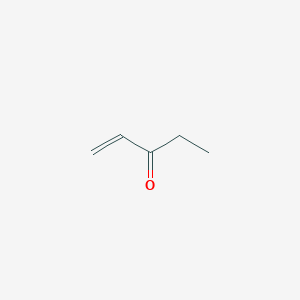 |
| --- | --- | --- | --- | --- | --- | --- |
| Evodiae Fructus | 56 | R24 | IMETHYLFU | 19462 | C6H8O | 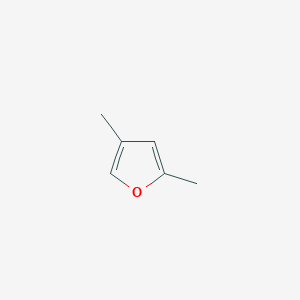 |
| Evodiae Fructus | 57 | R25 | yl-1,5-hexadie | 263169 | C8H14O2 | 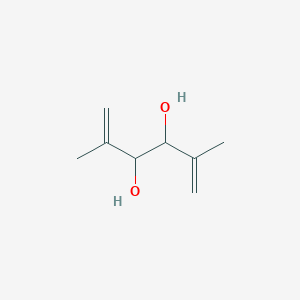 |

| Evodiae Fructus | 58 | R26 | l-4-methyl-4-v | 92138 | C15H26O | 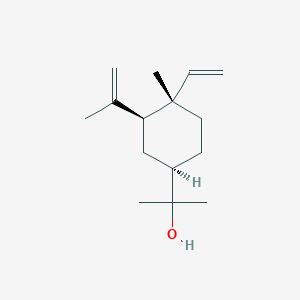 |
| --- | --- | --- | --- | --- | --- | --- |
| Evodiae Fructus | 59 | R27 | -31-norlanost- | 5319735 | C30H52O | 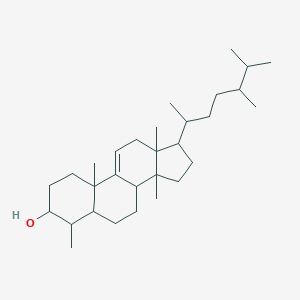 |
| Evodiae Fructus | 60 | R28 | formyl-7-meth | 189687 | C14H11NO3 | 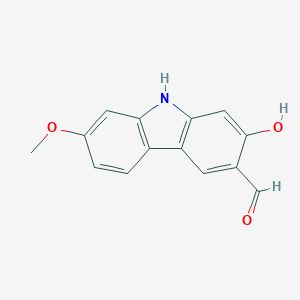 |

| Evodiae Fructus | 61 | R29 | ethyl-6-hepten | 144868 | C8H16O | 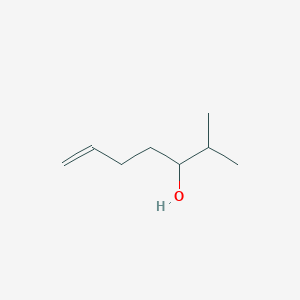 |
| --- | --- | --- | --- | --- | --- | --- |
| Evodiae Fructus | 62 | R30 | allyl-p-propen | 5316879 | C14H18O | 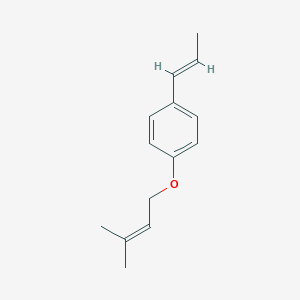 |
| Evodiae Fructus | 63 | R31 | 3691-11-0 | 94275 | C15H24 | 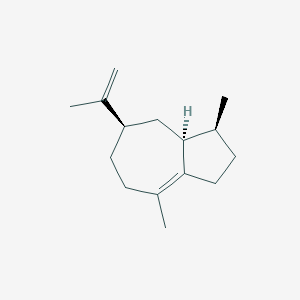 |

| Evodiae Fructus | 64 | R32 | cyclopent-2-en | 557256 | C7H10O | 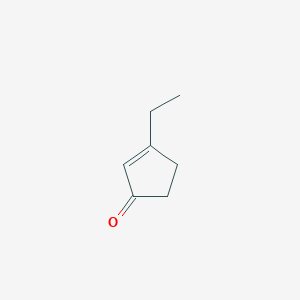 |
| --- | --- | --- | --- | --- | --- | --- |
| Evodiae Fructus | 65 | R33 | 3-Furaldehyde | 10351 | C5H4O2 | 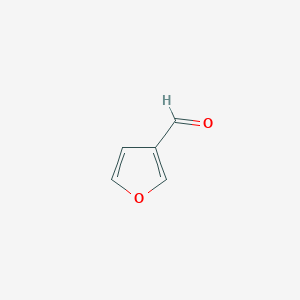 |
| Evodiae Fructus | 66 | R34 | xy-2-methylam | 667452 | C9H13NO2 | 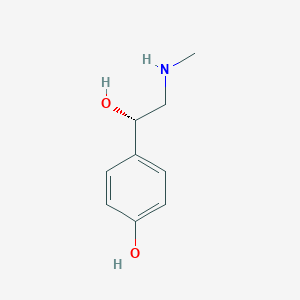 |

| Evodiae Fructus | 67 | R35 | pylcyclohex-2- | 92780 | C9H14O | 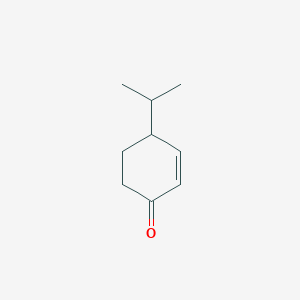 |
| --- | --- | --- | --- | --- | --- | --- |
| Evodiae Fructus | 68 | R36 | etradecadienoi | 151507 | C14H24O2 | 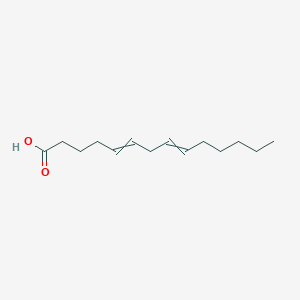 |
| Evodiae Fructus | 69 | R37 | methylbicyclo[ | 17868 | C10H16 | 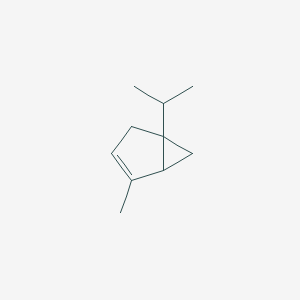 |

| Evodiae Fructus | 70 | R38 | Acetoxy-5-epi | 146156527 | C28H32O10 | 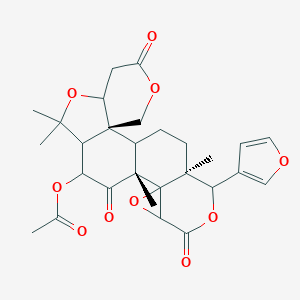 |
| --- | --- | --- | --- | --- | --- | --- |
| Evodiae Fructus | 71 | R39 | 6-OH-Luteolin | 5281642 | C15H10O7 | 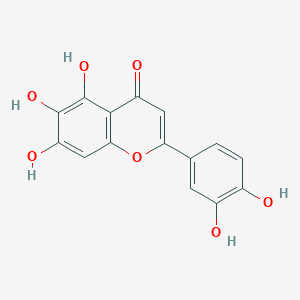 |
| Evodiae Fructus | 72 | R40 | ylidene-7-meth | 21160893 | C15H24O | 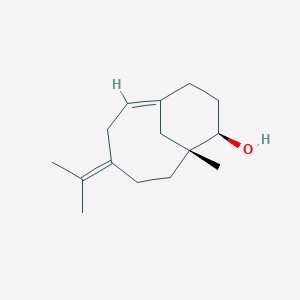 |

| Evodiae Fructus | 73 | R41 | ACID,BORNY | 637531 | C12H20O2 | 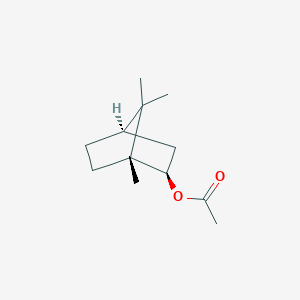 |
| --- | --- | --- | --- | --- | --- | --- |
| Evodiae Fructus | 74 | R42 | AI3-23133 | 89664 | C10H16O | 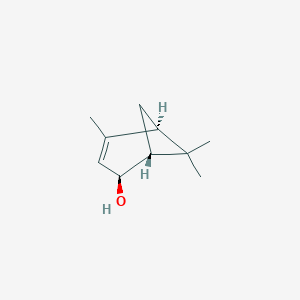 |
| Evodiae Fructus | 75 | R43 | lpha-Cubeben | 442359 | C15H24 | 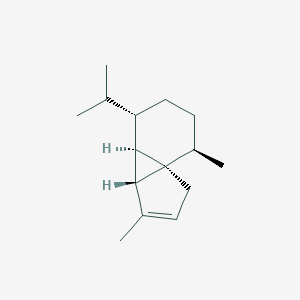 |

| Evodiae Fructus | 76 | R44 | lpha-humulen | 5281520 | C15H24 | 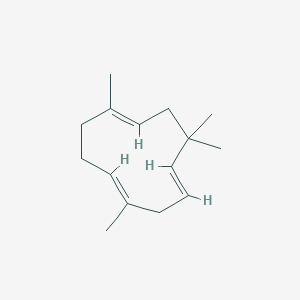 |
| --- | --- | --- | --- | --- | --- | --- |
| Evodiae Fructus | 77 | R45 | Arachidoside | 597207 | C16H16O6 | 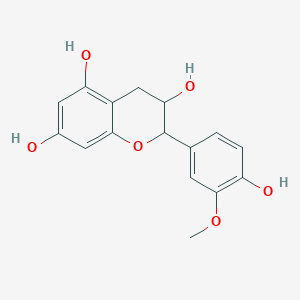 |
| Evodiae Fructus | 78 | R46 | enzylisovalera | 7651 | C12H16O2 | 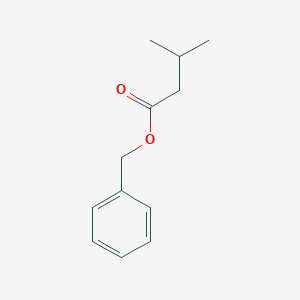 |

| Evodiae Fructus | 79 | CR1 | berberine | 2353 | C20H18NO4+ | 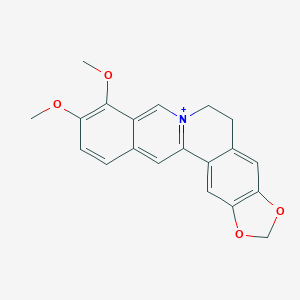 |
| --- | --- | --- | --- | --- | --- | --- |
| Evodiae Fructus | 80 | R47 | beta-Cubebene | 93081 | C15H24 | 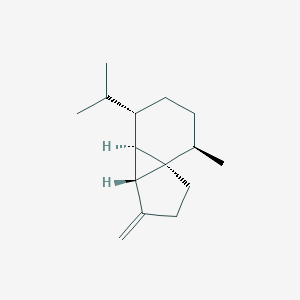 |
| Evodiae Fructus | 81 | R48 | beta-sitosterol | 222284 | C29H50O | 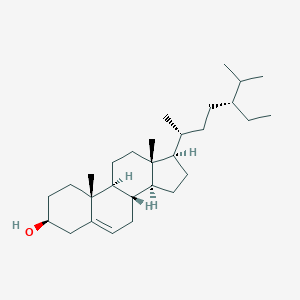 |

| Evodiae Fructus | 82 | R49 | BZM | 2345 | C14H12O2 | 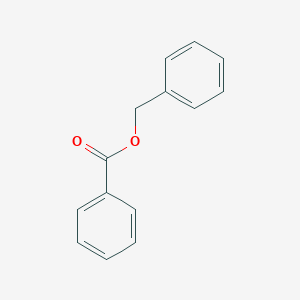 |
| --- | --- | --- | --- | --- | --- | --- |
| Evodiae Fructus | 83 | R50 | caffeine | 2519 | C8H10N4O2 | 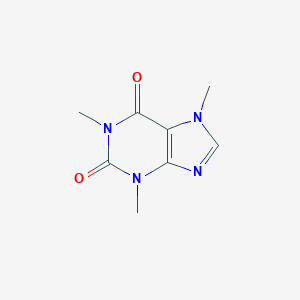 |
| Evodiae Fructus | 84 | R51 | CHEBI:7 | 443156 | C10H16 | 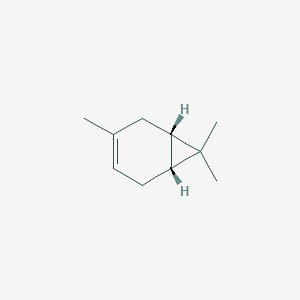 |

| Evodiae Fructus | 85 | R52 | Cinnamein | 5273469 | C16H14O2 | 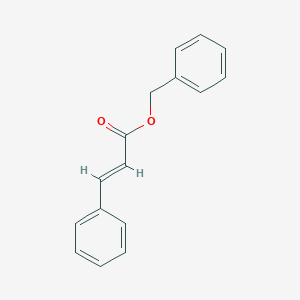 |
| --- | --- | --- | --- | --- | --- | --- |
| Evodiae Fructus | 86 | R53 | .-Elemenedias | 6431152 | C15H24 | 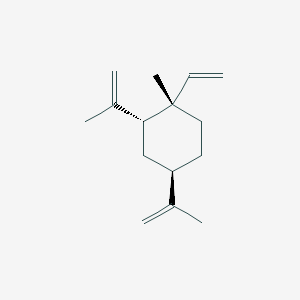 |
| Evodiae Fructus | 87 | R54 | citricacid | 311 | C6H8O7 | 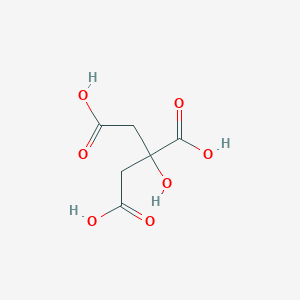 |

| Evodiae Fructus | 88 | R55 | Coumaran | 10329 | C8H8O | 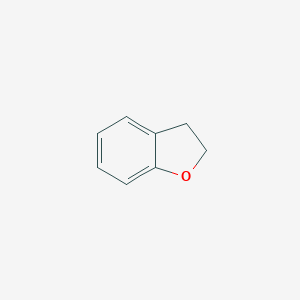 |
| --- | --- | --- | --- | --- | --- | --- |
| Evodiae Fructus | 89 | R56 | Cuminol | 325 | C10H14O | 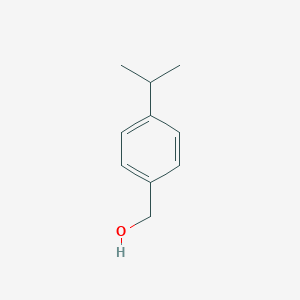 |
| Evodiae Fructus | 90 | R57 | lohexene,3-bu | 138093 | C10H18 | 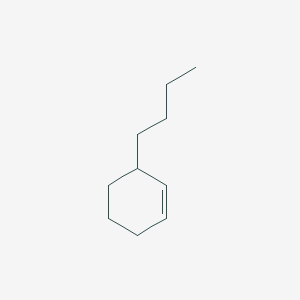 |

| Evodiae Fructus | 91 | R58 | D-Camphene | 92221 | C10H16 | 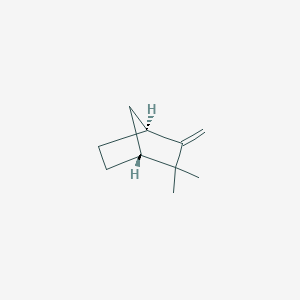 |
| --- | --- | --- | --- | --- | --- | --- |
| Evodiae Fructus | 92 | R59 | elta-amorphen | 10223 | C15H24 | 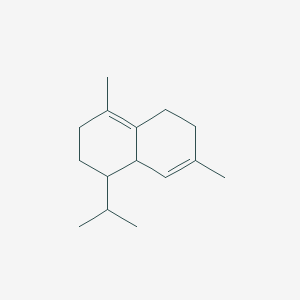 |
| Evodiae Fructus | 93 | R60 | ydrorutaecarpine | |  | 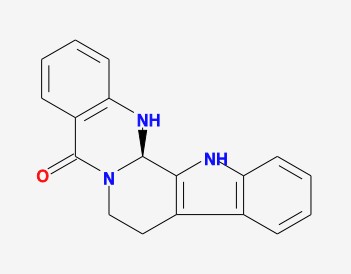 |

| Evodiae Fructus | 94 | R61 | methylanthrani | 6826 | C9H11NO2 | 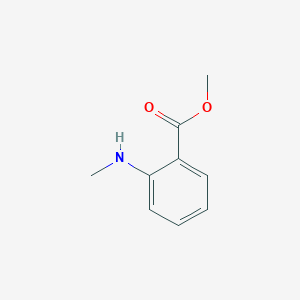 |
| --- | --- | --- | --- | --- | --- | --- |
| Evodiae Fructus | 95 | R62 | ELD | 5283387 | C18H35NO | 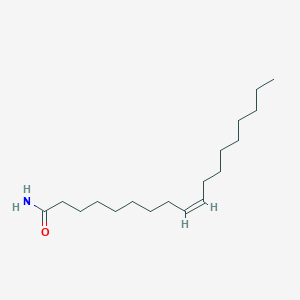 |
| Evodiae Fructus | 96 | R63 | EUG | 332 | C9H10O2 | 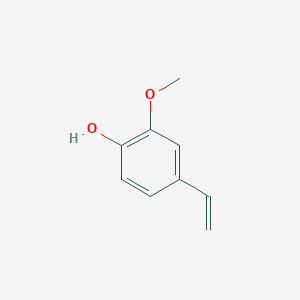 |

| Evodiae Fructus | 97 | R64 | eugenol | 3314 | C10H12O2 | 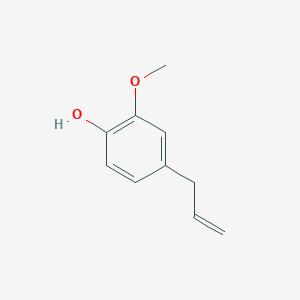 |
| --- | --- | --- | --- | --- | --- | --- |
| Evodiae Fructus | 98 | R65 | Evocarpine | 5317303 | C23H33NO | 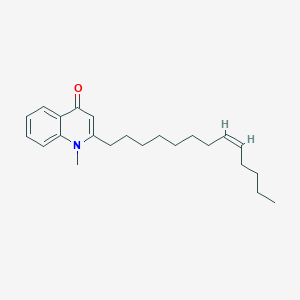 |
| Evodiae Fructus | 99 | R66 | Evodiamide | 189454 | C19H21N3O | 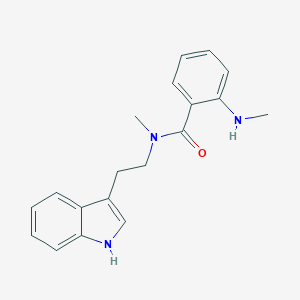 |

| Evodiae Fructus | 100 | R67 | Evodiamine | 442088 | C19H17N3O | 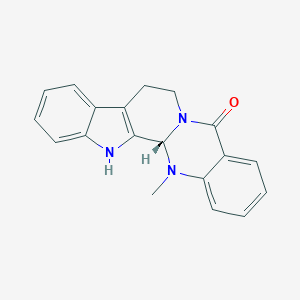 |
| --- | --- | --- | --- | --- | --- | --- |
| Evodiae Fructus | 101 | R68 | ranyl isobutyr | 6086514 | C14H24O2 |  |
| Evodiae Fructus | 102 | R69 | eranyl propion | 5355853 | C13H22O2 |  |

| Evodiae Fructus | 103 | R70 | germacrene | 9548705 | C15H24 |  |
| --- | --- | --- | --- | --- | --- | --- |
| Evodiae Fructus | 104 | R71 | Goshuyuamide | 5317827 | C19H19N3O |  |
| Evodiae Fructus | 105 | R72 | oshuyuamide | 5317828 | C19H17N3O2 |  |

| Evodiae Fructus | 106 | R73 | gossypetin | 5280647 | C15H10O8 |  |
| --- | --- | --- | --- | --- | --- | --- |
| Evodiae Fructus | 107 | R74 | vacridoneshlir | 5315835 | C19H18ClNO4 |  |
| Evodiae Fructus | 108 | R75 | Hemo-sol | 440917 | C10H16 |  |

| Evodiae Fructus | 109 | R76 | hexanal | 6184 | C6H12O |  |
| --- | --- | --- | --- | --- | --- | --- |
| Evodiae Fructus | 110 | R77 | droxyevodiam | 71307457 | C19H17N3O2 |  |
| Evodiae Fructus | 111 | R78 | isorhamnetin | 5281654 | C16H12O7 |  |

| Evodiae Fructus | 112 | CR4 | Isovanillin | 12127 | C8H8O3 |  |
| --- | --- | --- | --- | --- | --- | --- |
| Evodiae Fructus | 113 | R79 | l-carvone | 439570 | C10H14O |  |
| Evodiae Fructus | 114 | CR5 | limonin | 179651 | C26H30O8 |  |

| Evodiae Fructus | 115 | R80 | Linalool | 6549 | C10H18O |  |
| --- | --- | --- | --- | --- | --- | --- |
| Evodiae Fructus | 116 | R81 | L-Serin | 5951 | C3H7NO3 |  |
| Evodiae Fructus | 117 | R82 | MeODMT | 1832 | C13H18N2O |  |

| Evodiae Fructus | 118 | R83 | Methyleugeno | 7127 | C11H14O2 |  |
| --- | --- | --- | --- | --- | --- | --- |
| Evodiae Fructus | 119 | R84 | Moslene | 7461 | C10H16 |  |
| Evodiae Fructus | 120 | R85 | muurolene | 12306047 | C15H24 |  |

| Evodiae Fructus | 121 | R86 | Myrcene | 31253 | C10H16 |  |
| --- | --- | --- | --- | --- | --- | --- |
| Evodiae Fructus | 122 | R87 | laminobenzoy | 5319506 | C18H19N3O |  |
| Evodiae Fructus | 123 | R88 | Nantenin | 197001 | C20H21NO4 |  |

| Evodiae Fructus | 124 | R89 | thyl-4-methyle | 92313 | C15H24 |  |
| --- | --- | --- | --- | --- | --- | --- |
| Evodiae Fructus | 125 | R90 | erolidyl acetat | 5363426 | C17H28O2 |  |
| Evodiae Fructus | 126 | R91 | Nerylacetate | 7780 | C12H20O2 |  |

| Evodiae Fructus | 127 | R92 | Nevoli oil | 8635 | C8H9NO2 |  |
| --- | --- | --- | --- | --- | --- | --- |
| Evodiae Fructus | 128 | R93 | -Acetyl-p-cres | 15068 | C9H10O2 |  |
| Evodiae Fructus | 129 | R94 | o-N-methylbe | 308072 | C8H10N2O |  |

| Evodiae Fructus | 130 | R95 | o-Cymol | 10703 | C10H14 |  |
| --- | --- | --- | --- | --- | --- | --- |
| Evodiae Fructus | 131 | R96 | oleanolicacid | 191720 | C30H48O3 |  |
| Evodiae Fructus | 132 | R97 | palmitic acid | 985 | C16H32O2 |  |

| Evodiae Fructus | 133 | R98 | Perillyl alcoho | 10819 | C10H16O |  |
| --- | --- | --- | --- | --- | --- | --- |
| Evodiae Fructus | 134 | R99 | Physcion | 10639 | C16H12O5 |  |
| Evodiae Fructus | 135 | R100 | entha-1,5-dien | 519323 | C10H16O |  |

| Evodiae Fructus | 136 | R101 | PTL | 8063 | C5H10O |  |
| --- | --- | --- | --- | --- | --- | --- |
| Evodiae Fructus | 137 | CR3 | quercetin | 5280343 | C15H10O7 |  |
| Evodiae Fructus | 138 | R102 | rutaecarpine | 65752 | C18H13N3O |  |

| Evodiae Fructus | 139 | R103 | Rutalinidine | 51380995 | C15H17NO4 |  |
| --- | --- | --- | --- | --- | --- | --- |
| Evodiae Fructus | 140 | R104 | rutin | 5280805 | C27H30O16 |  |
| Evodiae Fructus | 141 | R105 | Safranal | 61041 | C10H14O |  |

| Evodiae Fructus | 142 | R106 | Sitogluside | 5742590 | C35H60O6 |  |
| --- | --- | --- | --- | --- | --- | --- |
| Evodiae Fructus | 143 | R107 | sitosterol | 222284 | C29H50O |  |
| Evodiae Fructus | 144 | R108 | SobrolA | 8434 | C9H10O3 |  |

| Evodiae Fructus | 145 | R109 | stearic acid | 5281 | C18H36O2 |  |
| --- | --- | --- | --- | --- | --- | --- |
| Evodiae Fructus | 146 | R110 | Syringaldehyd | 8655 | C9H10O4 |  |
| Evodiae Fructus | 147 | R111 | Terpilene | 7462 | C10H16 |  |

| Evodiae Fructus | 148 | R112 | trahydromagno | 5321851 | C18H22O2 |  |
| --- | --- | --- | --- | --- | --- | --- |
| Evodiae Fructus | 149 | R113 | Threonin | 6288 | C4H9NO3 |  |
| Evodiae Fructus | 150 | R114 | RIDECANON | 11622 | C13H26O |  |

| Evodiae Fructus | 151 | R115 | uracil | 1174 | C4H4N2O2 |  |
| --- | --- | --- | --- | --- | --- | --- |
| Evodiae Fructus | 152 | R116 | Veratral | 8419 | C9H10O3 |  |
| Evodiae Fructus | 153 | R117 | WLN:VHR | [240](http://pubchem.ncbi.nlm.nih.gov/summary/summary.cgi?cid=240) | C7H6O |  |

| Evodiae Fructus | 154 | R118 | WV | 6923516 | C11H12N2O2 |  |
| --- | --- | --- | --- | --- | --- | --- |
| Evodiae Fructus | 155 | R119 | ZINC0204097 | 11241545 | C15H26O |  |
| Evodiae Fructus | 156 | R120 | hydroevodiam | 9817839 | C19H15N3O |  |

| Evodiae Fructus | 157 | R121 | evodione | 624052 | C16H20O5 |  |
| --- | --- | --- | --- | --- | --- | --- |
| Evodiae Fructus | 158 | R122 | synephrine | 7172 | C9H13NO2 |  |
| Evodiae Fructus | 159 | CR2 | Obacunone | 119041 | C26H30O7 |  |

| Evodiae Fructus | 160 | R123 | -Methylmyrist | 151014 | C15H30O2 |  |
| --- | --- | --- | --- | --- | --- | --- |
| Evodiae Fructus | 161 | R124 | is-beta-Ocime | 5320250 | C10H16 |  |
